# Modulating Membrane Composition Alters Free Fatty Acid Tolerance in *Escherichia coli*

**DOI:** 10.1371/journal.pone.0054031

**Published:** 2013-01-21

**Authors:** Rebecca M. Lennen, Brian F. Pfleger

**Affiliations:** 1 Department of Chemical and Biological Engineering, University of Wisconsin-Madison, Madison, Wisconsin, United States of America; 2 United States Department of Energy Great Lakes Bioenergy Research Center, University of Wisconsin-Madison, Madison, Wisconsin, United States of America; University of Illinois at Urbana-Champaign, United States of America

## Abstract

Microbial synthesis of free fatty acids (FFA) is a promising strategy for converting renewable sugars to advanced biofuels and oleochemicals. Unfortunately, FFA production negatively impacts membrane integrity and cell viability in *Escherichia coli*, the dominant host in which FFA production has been studied. These negative effects provide a selective pressure against FFA production that could lead to genetic instability at industrial scale. In prior work, an engineered *E. coli* strain harboring an expression plasmid for the *Umbellularia californica* acyl-acyl carrier protein (ACP) thioesterase was shown to have highly elevated levels of unsaturated fatty acids in the cell membrane. The change in membrane content was hypothesized to be one underlying cause of the negative physiological effects associated with FFA production. In this work, a connection between the regulator of unsaturated fatty acid biosynthesis in *E. coli*, FabR, thioesterase expression, and unsaturated membrane content was established. A strategy for restoring normal membrane saturation levels and increasing tolerance towards endogenous production of FFAs was implemented by modulating acyl-ACP pools with a second thioesterase (from Geobacillus *sp.* Y412MC10) that primarily targets medium chain length, unsaturated acyl-ACPs. The strategy succeeded in restoring membrane content and improving viability in FFA producing *E. coli* while maintaining FFA titers. However, the restored fitness did not increase FFA productivity, indicating the existence of additional metabolic or regulatory barriers.

## Introduction

One of the most promising routes for producing renewable substitutes for petrodiesel makes use of intermediates derived from fatty acid biosynthesis in microbes such as *Escherichia coli*. By introducing a cytosolic acyl-acyl carrier protein (ACP) thioesterase, feedback inhibition of enzymes involved in fatty acid biosynthesis by acyl-ACP intermediates is released, increasing flux through fatty acid biosynthesis [1]–[3]. The released free fatty acids (FFA) can either be separated from the culture medium and catalytically decarboxylated to alkanes [4], [5], or they can be directed into heterologous pathways that produce products that include fatty acid ethyl esters, fatty alcohols, alkanes, olefins, methyl ketones, and polyhydroxyalkanoates [6]–[10].

Past reports have indicated that heterologous expression of the acyl-ACP thioesterase from *Umbellularia californica* (BTE) in *E. coli* results in greatly elevated levels of unsaturated and cyclopropane phospholipids, which are derived from unsaturated acyl-ACPs [11], [12]. BTE belongs to the FatB family of plant acyl-ACP thioesterases, and has specificity for predominantly saturated C_12_ acyl-ACPs (∼60–70% of FFAs), while also hydrolyzing unsaturated C_12_ (∼10%), saturated C_14_ (∼10%), and unsaturated C_14_ (∼10%) [12]. The heightened unsaturated membrane lipid content was postulated to result from altered long-chain acyl-ACP pools caused by BTE-mediated depletion of saturated acyl-ACP. Altered membrane content has been observed in cells expressing other thioesterases with the changes directly correlated to thioesterase substrate specificity. For instance, expression of the FatA type thioesterase from *Helianthus annuus* in *E. coli*, which predominantly cleaves unsaturated C_16_ and C_18_ acyl-ACPs, increased the saturated phospholipid acyl content by approximately 5% [13]. Overexpression of a cytosolic form of *E. coli* thioesterase I (TesA′), which was reported to generate a FFA distribution of approximately 54% unsaturated and 46% saturated, also resulted in a 7.5% reduction of unsaturated and cyclic phospholipid acyl group content [1].

**Figure 1:**
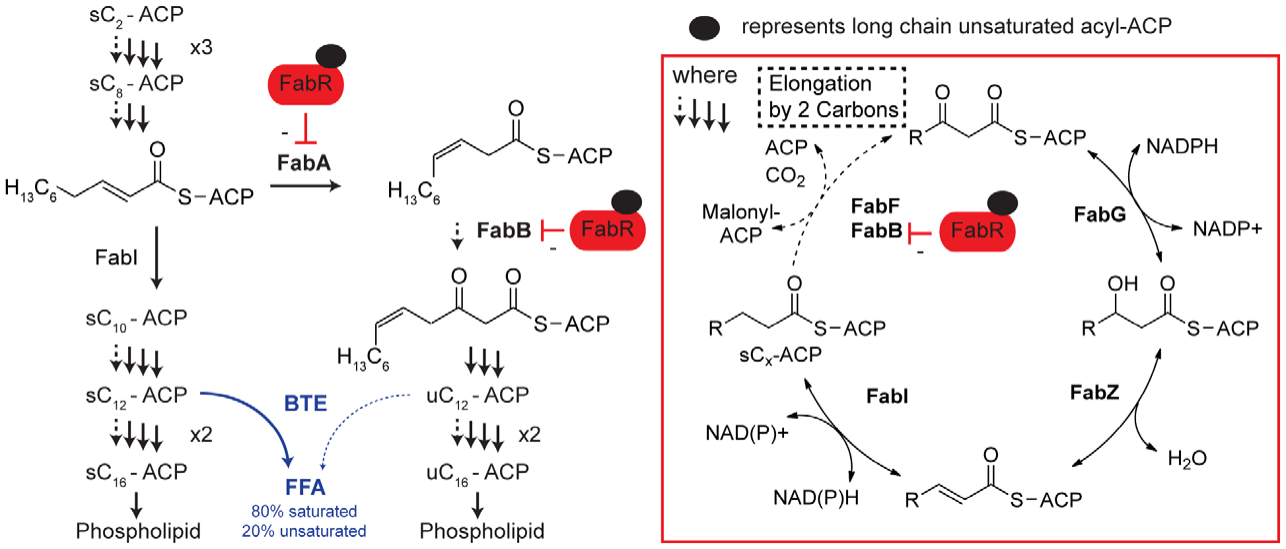
Unsaturated fatty acid biosynthesis and regulation. Unsaturated fatty acid biosynthesis begins with the isomerization of *trans*-2-decenoyl-ACP to *cis*-3-decenoyl-ACP by FabA. Instead of being reduced, this intermediate is condensed with malonyl-ACP by FabB. The resulting unsaturated β-ketoacyl-ACP is processed analogous to its saturated counterpart until unsaturated C_16_ and C_18_ acyl-ACPs are made and incorporated into phospholipids. Unsaturated fatty acid biosynthesis is feedback-inhibited at the transcriptional level by FabR, which exhibits increased repression of transcription of *fabA* and *fabB* when bound to enoyl-ACP species [16], [17]. Expression of a thioesterase cleaves acyl-ACPs to generate FFA. BTE expression preferentially cleaves saturated C_12_-acyl-ACPs (solid blue arrow) and minorly cleaves unsaturated C_12_- (dashed blue arrow) and saturated and unsaturated C_14_-acyl-ACPs, thereby depleting saturated, long chain acyl-ACPs, the key regulatory signal for controlling fatty acid biosynthesis. As a result, flux through the saturated (prior to C_10_) and unsaturated pathway increases. Inset: the four arrows represent the elongation (FabB/FabF) (dashed arrow) in which the acyl chain represented by R grows by 2 carbons, ketoreduction (FabG), dehydration (FabZ), and enoyl reduction (FabI) reactions that comprise one round of fatty acid elongation and reduction.

In prior work, we observed strong decreases in expression of *fabA* and *fabB*, genes required for unsaturated fatty acid biosynthesis, in cultures expressing BTE compared to cultures expressing a non-functional thioesterase with a catalytic histidine mutagenized to an alanine, BTE-H204A [11]. This was concomitant with increased unsaturated C_16_–C_18_ fatty acid content associated with membrane phospholipids [11], [12]. FabA, which catalyzes formation of the *cis* double bond in elongating acyl chains at the C_10_ chain length, and FabB, which is essential for condensing *cis*-3-decenoyl-ACP with malonyl-ACP, are both regulated at the transcriptional level by DNA binding of FabR to their promoter region [14], [15] (**Figure 1**). FabR binds DNA and both saturated and unsaturated acyl-ACPs, with a stronger affinity for DNA when bound to unsaturated acyl-ACPs [16], [17]. FabR thus plays a key role in modulating unsaturated membrane lipid content [16]. Due to this mechanism of repression, we hypothesized that expression of BTE, which primarily cleaves saturated acyl-ACPs, was enriching the acyl-ACP pool in unsaturated acyl-ACPs (**Figure 1**). The high proportion of unsaturated acyl-ACPs increased FabR-mediated repression, reducing transcription of *fabA* and *fabB*, but this repression was insufficient to prevent a highly elevated unsaturated membrane lipid content. The altered membrane composition would be expected to affect membrane properties, and could be responsible (in full or in part) for the negative phenotypes associated with endogenous FFA production (e.g. increased membrane depolarization and induction of membrane stress responses) [11]. The inability of cells to effectively regulate membrane fatty acid saturation as a result of BTE expression could become a selective pressure against endogenous FFA production and therefore a potential source of strain instability at industrial scale.

In this study, we investigated the impact of BTE expression on unsaturated membrane lipid content, cell lysis, FFA titers, and *fabA* and *fabB* expression levels in the absence of FabR, eliminating the mechanism for feedback repression of unsaturated fatty acid biosynthesis. Higher levels of *fabA* and *fabB* expression were observed, correlated with a greatly elevated unsaturated membrane lipid content, a highly exacerbated degree of cell lysis, and depressed FFA titers, underscoring the importance of FabR-mediated control of unsaturated fatty acid biosynthesis toward tolerance of endogenous FFA production. As a demonstration of the ability to modulate membrane lipid composition by acyl-ACP thioesterase selection, a thioesterase from *Geobacillus sp.* Y412MC10 (GeoTE) which was reported to hydrolyze a high percentage of unsaturated medium chain-length FFAs [18], was expressed by itself and in combination with BTE. Expression of GeoTE both alone and in tandem with BTE reduced the membrane unsaturated fatty acid content, relieved transcriptional repression of *fabA* and *fabB*, and decreased the population of lysed cells while maintaining total FFA titers. Our findings emphasize the importance of membrane physiology in biofuel producing strains and illustrate how unintended consequences can arise when native metabolism is manipulated.

## Materials and Methods

### Chemicals, reagents, enzymes, and oligonucleotide primers

All chemicals were purchased from Fisher Scientific (Pittsburgh, PA) unless indicated otherwise. Cloning and PCR reagents were purchased from New England Biolabs (Ipswich, MA), Fermentas (Glen Burnie, MD), Promega (Madison, WI), and Qiagen (Valencia, CA). Oligonucleotides (**Table S1**) were purchased from Integrated DNA Technologies (Coralville, IA).

### Strain construction

Bacterial strains and plasmids used in this study are listed in **Table 1**. The background strain used in this study is RL08ara, a strain derived from RL08 (K-12 MG1655 Δ*fadD* Δ*araBAD*, [4]) with the additional deletion of *araFGH* encoding a high-affinity arabinose transporter, and replacement of the native promoter of *araE*, a low-affinity arabinose transporter, with a constitutive promoter. These modifications were previously shown to generate homogeneous induction from the P_BAD_ promoter across a population of cells, and to enable titration of arabinose to modulate gene expression from a P_BAD_ promoter [19].

**Table 1:** Strains and plasmids used in this study.

Strain/plasmid	Relevant genotype/property^a^	Source/Reference
**Strains**		
DH10B	F^−^ *mcr*A Δ(*mrr-hsd*RMS-*mcr*BC) φ80*lac*ZΔM15 Δ*lac*X74 *rec*A1 *end*A1 *ara*D139 Δ(*ara*, *leu*)7697 *gal*U *gal*K λ^−^ *rps*L *nup*G	Invitrogen
DH5α	*fhuA2* Δ(*argF-lacZ*)*U169 phoA glnV44* Φ*80* Δ(*lacZ*)*M15 gyr*A96 *rec*A1 *rel*A1 *end*A1 *thi*-1 *hsd*R17	Invitrogen
BW25113	*lacI^q^ rrnB3* F- *Δ(araD-araB)567 ΔlacZ4787*(::rrnB-3) *λ^−^ rph*-1 Δ(*rha*D-*rha*B)568 *hsd*R514	[21]
BW27269	BW25113 *araFGH*::*kan903*	[19]
BW27270	BW25113 Φ(Δ*araEp kan* P_CP18_-*araE*)	[19]
RL08	K-12 MG1655 Δ*fadD* Δ*araBAD*	[4]
RL14	RL08 *araFGH*::*kan*	This work
RL15	RL08 Δ*araFGH*	This work
RL16	RL08 Δ*araFGH* Φ(Δ*araEp kan* P_CP18_-*araE*)	This work
RL08ara	RL08 Δ*araFGH* Φ(Δ*araEp* P_CP18_-*araE*)	This work
JW3935-4	BW25113 Δ*fabR751::kan*	[21]
RL17	RL08ara *fabR::kan*	This work
RL18	RL08ara Δ*fabR*	This work
**Plasmids**		
pCP20	carries yeast FLP recombinase under constitutive promoter, pSC101 origin, λ cI857+, λ pR Repts, AmpR, Cm^R^	[20]
pBAD33	P_BAD_ promoter, pACYC origin, Cm^R^	[32]
pBAD18	P_BAD_ promoter, pBR322 origin, Amp^R^	[32]
pBAD33-BTE	pBAD33 carrying BTE under P_BAD_ control, Cm^R^	[4]
pBAD33-BTE-H204A	pBAD33 carrying BTE-H204A under P_BAD_ control, Cm^R^	[4]
pTrc99A	P_trc_ promoter, pBR322 origin, Amp^R^	[33]
pTrc99A-BTE	pTrc99A carrying BTE under P_trc_ control, Amp^R^	[11]
pTrc99A-BTE-H204A	pTrc99A carrying BTE-H204A under P_trc_ control, Amp^R^	[11]
pBAD33*	pBAD33 with *araC*-C280* mutation	This work
pBAD18-GeoTE	pBAD18 carrying *Geobacillus* sp. TE under P_BAD_ control, Amp^R^	This work
pBAD18-GeoTE-H173A	pBAD18 carrying *Geobacillus* sp. TE with H173A mutation under P_BAD_ control, Amp^R^	This work
pBAD18-ClosTE	pBAD18 carrying *Clostridium thermocellum* TE under P_BAD_ control, Amp^R^	This work
pBAD18-ClosTE-H171A	pBAD18 carrying *Clostridium thermocellum* TE with H171A mutation under P_BAD_ control, Amp^R^	This work
pBAD33*-fabR	pBAD33* carrying *fabR* under P_BAD_ control, Cm^R^	This work

a Abbreviations: Amp, ampicillin; Cm, chloramphenicol; R, resistance; ts, temperature sensitive.

Strain RL08ara was constructed by sequential P1 phage transduction using lysates harboring Φ(Δ*araEp kan* P_cp8_-*araE*) and *araFGH*::*kan* loci from strains BW27271 and BW27269, respectively [19]. Antibiotic resistance genes were removed after each transduction using pCP20 [20]. Plasmid pCP20 was removed by repeated elevated temperature cures at 43°C, and the presence of all desired FRT-site containing loci were confirmed by colony PCR using primers 1–8 (**Table S1**). Promoter replacement of *araE* was verified sequentially following both transduction and elevated temperature cure of pCP20.

Strain RL08ara Δ*fabR* was constructed by P1 phage transduction of the *fabR*::*kan* cassette from strain JW3935-4 [21]. The kanamycin resistance gene was removed using pCP20 as described above, and all FRT-site containing loci were confirmed by colony PCR using primers 9–10.

### Gene synthesis and plasmid construction

Codon-optimized genes encoding *Geobacillus sp.* Y412MC10 (recently renamed *Paenibacillus* sp. Y412MC10, GenBank genome accession number CP001793.1) acyl-ACP thioesterase (GeoTE), and *Clostridium thermocellum* acyl-ACP thioesterase (ClosTE, GenBankABN54268) were synthesized by GeneArt (Life Technologies, Regensburg, Germany). Ribosome binding sites (RBS) predicted to lead to a high rate of translation (∼25000 au) were designed using the Ribosome Binding Site Calculator [22]. The RBS and flanking restriction sites at the 3′ (XmaI) and 5′ (HindIII) termini were added to the final sequence to be synthesized. The ordered gene sequences are shown in **Figure S1**. A site-directed mutant was also ordered from GeneArt, following alignment of GeoTE and ClosTE with BTE (**Figure S2**), which identified a conserved catalytic histidine at positions 173 and 171, respectively. The full synthesized sequences were amplified by PCR using primers 13–14 for GeoTE, and 15–16 for ClosTE (3′ hexahistidine tags were added), digested with XmaI and HindIII, and ligated into plasmid pBAD18 to generate plasmids pBAD18-GeoTE, pBAD18-GeoTE-H173A, pBAD18-ClosTE, and pBAD18-ClosTE-H171A.

The *fabR* gene was amplified by PCR from MG1655 genomic DNA with flanking 5′ (XmaI) and 3′ (HindIII) restriction sites and an artificial RBS generated from the RBS Calculator forward design tool, using primers 17–18. The PCR product was subsequently digested with XmaI/HindIII, and ligated into pBAD33*, an arabinose inducible expression vector harboring a mutated *araC* (C280*) that enables co-induction of IPTG-inducible and arabinose-inducible promoter systems [23]. This vector was generated by PCR using primers 11–12 with template pBAD33, which introduced the C280* mutation and an XhoI restriction site at the 5′ and 3′ ends. The PCR product was then digested with XhoI and ligated to form plasmid pBAD33*.

### Cell cultivation

Cell cultures used in viability, RNA quantification, and fatty acid production experiments were grown at 37°C with shaking (250 rpm) in 250 ml shake flasks with a 4× headspace in Difco LB medium (BD, Franklin Lakes, NJ) supplemented with 0.4% v/v glycerol. Chloramphenicol was added to a concentration of 34 µg/ml in strains harboring pBAD33* derived plasmids. Ampicillin was added to a concentration of 50 µg/ml in strains harboring pBAD18 derived plasmids, and 100 µg/ml in strains harboring pTrc99A derived plasmids. Cultures were induced at OD_600_ 0.2 (except where indicated otherwise) with 0.2% arabinose for expression of genes on pBAD33, pBAD33*, and pBAD18, and 50 µM IPTG for expression of genes on pTrc99A. Three biological replicates of each strain were grown from overnight cultures inoculated with independent colonies on streak plates or fresh plasmid transformation plates.

### SYTOX flow cytometry assays

To assess cell permeability, cell pellets were collected and stained by addition of 1 µL of 5 mM SYTOX Green in DMSO (Invitrogen) and measured by flow cytometry as described in [11], [24]. Two distinct populations were evident from the green fluorescence histograms, allowing a logarithmic-scale green fluorescence intensity of 420 to serve as the cut-off between cells counted as intact (less than or equal to 420) and non-intact (greater than 420).

### Cell viability measurements from plate counts

Volumes of cell culture were serially diluted in phosphate buffered saline (PBS) (137 mM NaCl, 27 mM KCl, 10 mM Na_2_HPO_4_, 2 mM KH_2_PO_4_, pH 7.4) and spread onto LB agar plates (containing no antibiotics) at indicated times. Individual colonies were counted after overnight incubation at 37°C and additional overnight incubation as specified.

### Fatty acid extraction and analysis

Total fatty acids were extracted from cell cultures and methylated by acid catalysis as previously described [4]. In separate analyses, bound fatty acids were extracted and methylated by base catalysis. Samples were extracted from 2.5 mL of cell culture spiked with 5 µl of 10 mg/mL heptadecanoic acid (Fluka) in ethanol and 10 µL of 2.5 mg/mL 1,2-dipentadecanoyl*-sn*-glycero-3-phosphoethanolamine (Avanti Polar Lipids, Alabaster, AL) in chloroform with 5 mL of 1∶1 chloroform∶methanol, vortexed thoroughly, and centrifuged at 1000×*g* for 10 min. The aqueous upper layer and interfacial cell debris was removed, and the bottom organic layer was evaporated to dryness under a nitrogen stream. To the dried residue, 0.5 ml of 0.5 M sodium methoxide in methanol (Sigma) was added, and the reactions were allowed to proceed at 50°C for 10 minutes [25]. To quench the reaction, 0.1 ml of glacial acetic acid was added, followed by 5 ml of deionized water. Fatty acid methyl esters (FAME) were extracted twice into 0.5 ml of hexane, and the collected hexane layers were quantified for total fatty acids. Gas chromatography/mass spectrometry (GC/MS) analysis and peak identification and quantification was performed on a model 7890 Agilent GC with a model 5975 mass spectrometer as described previously [4]. Average fatty acid concentrations from biological triplicate cultures were determined by normalizing to recovered FFA internal standards (pentadecanoic and heptadecanoic acid) from acid-catalyzed methylations, and from the recovered fatty acids derived from the phospholipid internal standard in base-catalyzed methylations (heptadecanoic acid was added for verification that FFA were not being methylated).

### RNA extraction and qPCR

From shake flask cultures expressing combinations of pBAD33-BTE-H204A, pBAD33-BTE, pBAD18-GeoTE, and pBAD18-GeoTE-H173A, cells were collected in mid-log phase (OD ∼0.8, 3.25 hours post-inoculation) and in early stationary phase (OD varied between 0.6 to 4.6, 5.25 hours post-inoculation) by spinning down 0.8 ml and 0.333 ml of culture, respectively, centrifuging at 16,000×*g* for 1 min, aspirating off the supernatant, and flash freezing the cell pellet in a dry ice/ethanol bath. Cell pellets were stored at −80°C. To extract RNA, cell pellets were resuspended in 100 µl of TE buffer, pH 8.0, containing 400 µg/ml lysozyme and incubated for 5 minutes. From this point forward, the Qiagen RNeasy Plus Kit was used according to the manufacturer's instructions. For shake flask cultures expressing combinations of pTrc99A-BTE, pTrc99A-BTE-H204A, pBAD33*, and pBAD33*-fabR in strains RL08ara and RL08ara Δ*fabR*, approximately 0.7 OD_600_-ml of cells (i.e. 0.7 ml of OD_600_ 1.0) were collected in early stationary phase (4.6 h post-inoculation) and centrifuged at 16,000×*g* for 1 min. The supernatant was removed by aspiration and cell pellets were flash frozen in a dry ice/ethanol bath prior to storage at −80°C. RNA was extracted and purified as described above, with the exception of using the Qiagen RNeasy Kit.

Following RNA clean-up, contaminating DNA was removed using the DNA-free™ Kit (Applied Biosystems, Carlsbad, CA) according to the manufacturer's instructions. The absence of DNA was confirmed by using 0.5 µl of each RNA sample as template in a PCR using primers 25–26 (**Table S1**). First-strand cDNA synthesis was performed for the first set of shake flask cultures using the Promega GoScript™ Reverse Transcription System according to the manufacturer's instructions, with 0.5 µg of RNA, random primers, 2.5 mM MgCl_2_, and a 1 hour extension time at 42°C. First-strand cDNA synthesis for the second set of shake flask cultures was performed using the Bio-Rad iScript™ Reverse Transcription Supermix (Hercules, CA) for RT-qPCR with 1 µg RNA template. The presence of cDNA was confirmed in each reaction using 0.5 µl of each sample as template as described above for confirming the lack of DNA contamination in RNA samples. To quantify the relative expression of *fabA*, *fabB*, and *fabR* transcript in each sample, qPCR reactions were set up using Fermentas Maxima SYBR Green master mix was used according to the manufacturer's directions, with 2.0 µl of 5-fold diluted cDNA in water was used as template, and primers 19–24 (**Table S1**). SYBR green fluorescence was monitored with a Bio-Rad CFX96 optical reaction module. Cycle quantification (C_q_) values were calculated by Bio-Rad CFX Manager software, and relative expression values to the double negative controls were calculated as 2^−ΔCq^.

## Results

### Effect of *fabR* deletion on unsaturated membrane-bound fatty acids

To verify that the elevated levels of unsaturated C_16_ and C_18_ fatty acids found in BTE expressing *E. coli* cultures [11], [12] were located in membrane lipids, we compared fatty acid methyl ester (FAME) profiles prepared by acid and base catalysis. Acid catalysis methylates both FFA and bound fatty acids in phospholipids and other species, whereas base catalysis methylates only bound fatty acids. FAME samples were prepared from replicate cultures of *E. coli* RL08ara harboring pBAD33-BTE and pBAD18-GeoTE-H173A. The difference between the total and bound fatty acid profiles provided the FFA profile, which demonstrated that BTE does not significantly cleave C_16_ to C_18_ chain length fatty acids (**Figure S3**).

**Figure 2:**
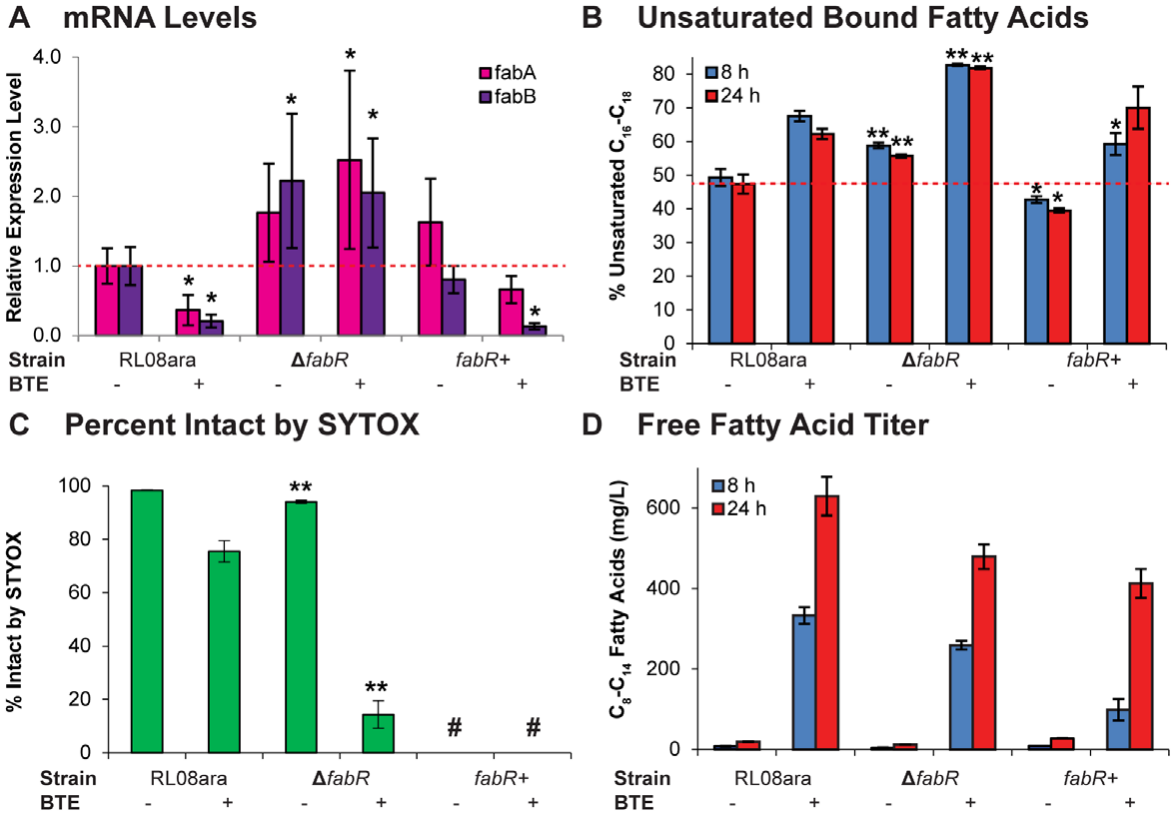
Connection between FabR and unsaturated membrane content in FFA producing *E. coli*. a.) Transcript levels of *fabA* and *fabB* determined by qPCR on samples harvested 4.6 hours after inoculation were normalized to RL08ara harboring pTrc99A-BTE-H204A and pBAD33*. In strains carrying *fabR* (RL08ara, pBAD33*, labeled RL08ara, and RL08ara Δ*fabR*, pBAD33*fabR, labeled *fabR+*), expression of BTE (+) reduced both *fabA* and *fabB* levels. In Δ*fabR* strains (RL08ara Δ*fabR*, pBAD33*) levels of *fabB* were increased in cells expressing both BTE-H204A (−) and BTE (+). Error bars represent propagated standard errors about the mean of biological triplicate samples. P-values were calculated by a t-test of the C_q_ values of *fabA* or *fabB* in strain RL08ara BTE-H204A Samples with P-values less <0.05 were marked with an asterisk. b.) The percentage of unsaturated C_16_–C_18_ and cyclopropane (C_17Δ_) fatty acids were calculated from fatty acid samples extracted from cultures 8 hours post-inoculation. Expression of BTE dramatically increased unsaturated content in strains harboring chromosomal *fabR* (RL08ara), and further increased unsaturated content in the Δ*fabR* strain. Overexpression of *fabR* on a plasmid in the Δ*fabR* strain restored unsaturated content to a lower level than present in RL08ara. Error bars represent standard errors about the mean of biological triplicate samples. * = P-value<0.05, ** = P-value<0.01 compared against RL08ara BTE^−^ for BTE^−^ cultures or RL08ara BTE^+^ for BTE^+^ cultures at the same sampling time. c.) The percentage of intact cells were calculated from histograms of cells stained with SYTOX Green. In BTE-H204A-expressing cultures, deletion of *fabR* had little effect on percent intact cells. In BTE-expressing cultures, a dramatic decrease in the number of intact cells was observed as a result of deletion of *fabR*. Samples from *fabR*+ cells exhibited altered histograms and were not quantified (#). Error bars represent standard errors about the mean of biological triplicate samples. ** = P-value<0.01 compared against RL08ara BTE^−^ for BTE^−^ cultures or RL08ara BTE^+^ for BTE^+^ cultures. d.) Effect of *fabR* deletion on C_8_–C_14_ (predominantly free) fatty acid titer produced in BTE-expressing cultures. Reduced titers were observed at both 8 h and 24 h growth in the Δ*fabR* strain. Error bars represent standard deviations about the mean of biological triplicate samples.

If our hypothesis (FabR mediated repression of *fabA*/*fabB* is insufficient to overcome the BTE-mediated alteration of acyl-ACP pools) is correct, deletion of *fabR* should result in both increased levels of *fabA* and *fabB*, as well as increased unsaturated membrane content (a phenotype that should be further amplified by expression of BTE). To test this hypothesis, the levels of *fabA* and *fabB* transcript in early stationary phase (approximately 4.5 hours after inoculation) were quantified by qPCR (**Figure 2a**). Sampled cultures included RL08ara or RL08ara Δ*fabR* harboring combinations of plasmids (pTrc99A-BTE-H204A or pTrc99A-BTE, and pBAD33* or pBAD33*-fabR) that were induced at an OD_600_ between 0.05 and 0.12. Strain RL08ara/pTrc99A-BTE exhibited statistically significant (P<0.05) decreased levels of *fabA* and *fabB* relative to strain RL08ara/pTrc99A-BTE-H204A, consistent with previous results comparing BTE- and BTE-H204A-expressing strains under different growth conditions [11]. Strains with deletions in *fabR* exhibited heightened levels of *fabA* and *fabB* expression in both BTE-H204A (*fabA*, P = 0.069; *fabB*, P = 0.033) and BTE-expressing strains (*fabA*, P = 0.034; *fabB*, P = 0.031) relative to RL08ara/pTrc99A-BTE-H20A. The fold-change in *fabA* and *fabB* expression in response to *fabR* deletion was larger in the BTE-expressing strains than in the BTE-H204A-expressing strains, consistent with the hypothesis of an increased degree of FabR-mediated repression that is present when BTE is expressed.

Fatty acid titers were analyzed following extraction 8 h and 24 h post-inoculation. As predicted by the increased levels of *fabA* and *fabB*, strain RL08ara Δ*fabR*/pTrc99A-BTE-H204A also exhibited a higher percentage of unsaturated C_16_-C_18_ fatty acids at 8 h (58.8±0.8%) than RL08ara/pTrc99A-BTE-H204A (49.3±2.5%), and strain RL08ara Δ*fabR*/pTrc99A-BTE exhibited an even larger increase (82.7±0.3%) than RL08ara/pTrc99A-BTE (67.5±1.5%) (**Figure 2b**). Fatty acid compositions followed the same trend when extracted 24 h post-inoculation. These percentages include C_17_ cyclopropane fatty acids, which are derived from methylation across the double bond of 16∶1 fatty acids in phospholipids by cyclopropane fatty acid synthase (Cfa).

Given the impact of FabR on regulating *fabA* and *fabB* expression in the presence of BTE, we hypothesized that overexpression of FabR could increase repression and restore the unsaturated fatty acid membrane content toward original levels. Measurement of relative *fabR* transcript levels by qPCR indicated a 106±65 (P<0.001) and 82±51 (P<0.001) fold increase in strain RL08ara Δ*fabR*/pTrc99A-BTE-H204A and RL08ara Δ*fabR*/pTrc99A-BTE overexpressing *fabR* on pBAD33*-fabR, respectively, over the native levels of *fabR* expression in strain RL08ara harboring pBAD33*. Despite growth defects in strains carrying pBAD33*-fabR, overexpression of *fabR* restored decreased levels of *fabB* in RL08ara Δ*fabR* (**Figure 2a**), and also restored unsaturated C_16_–C_18_ fatty acid levels in RL08ara Δ*fabR* expressing either BTE-H204A or BTE to similar or lower levels as those present in RL08ara (**Figure 2b**). While not further considered in this study, *fabR* overexpression at lower levels may be a viable strategy for reversing the increased unsaturated fatty acid membrane composition resulting from expression of acyl-ACP thioesterases with predominantly saturated acyl-ACP substrate specificity.

### Effect of *fabR* deletion on cell membrane integrity

Cells from the same cultures described in **3.1** were collected 8 h post-inoculation and stained with SYTOX Green. The green fluorescence of individual cells in the population was measured by flow cytometry. SYTOX Green is ordinarily impermeable to intact inner cell membranes, but results in a bright green fluorescence upon nucleic acid binding in cells with non-intact inner membranes [26]. RL08ara/pTrc99A-BTE-H204A was 98.4±0.2 percent intact while RL08ara Δ*fabR*/pTrc99A-BTE-H204A exhibited only a slight reduction to 94.0±0.6 percent intact (**Figure 2c**). However, RL08ara/pTrc99A-BTE was 75.5±4.0 percent intact while RL08ara Δ*fabR*/pTrc99A-BTE had a dramatic reduction to 14.4±5.2 percent intact (**Figure 2c**). Therefore deletion of *fabR* has a specifically deleterious effect on cell membrane integrity under conditions of fatty acid overproduction.

### Effect of *fabR* deletion on FFA production

The negative physiological consequences of *fabR* deletion manifested in FFA production of BTE-expressing strains. RL08ara/pTrc99A-BTE exhibited higher titers of C_8_–C_14_ fatty acids at both 8 h and 24 h post-inoculation compared to RL08ara Δ*fabR*/pTrc99A-BTE (**Figure 2d**).

### Selection and expression of a predominant enoyl-ACP thioesterase

BTE is in the FatB family of plant acyl-ACP thioesterases, all of which exhibit predominantly saturated acyl-ACP substrate specificities [27]. Conversely, the FatA family of plant acyl-ACP thioesterases primarily hydrolyze *cis*-18:1Δ9-ACP [27], and no FatA thioesterase has been characterized in the literature that can hydrolyze medium-chain-length unsaturated species as a significant portion of its product profile. Recently, a number of bacterial acyl-ACP thioesterases were characterized by heterologous expression in a *fadD* deficient strain of *E. coli* [18]. Thioesterases from *Clostridium thermocellum* and *Geobacillus sp.* Y412MC10 produced greater quantities of unsaturated than saturated C_12_–C_14_ species and lower levels of octanoic acid than other tested thioesterases. Therefore codon-optimized genes encoding these acyl-ACP thioesterases (hereafter referred to as ClosTE and GeoTE) were chemically synthesized. Catalytic histidines were identified that aligned with His-204 and shared a NXHVNN motif previously identified by alignment of plant FatA and FatB thioesterases [28], with the exception of GeoTE having a leucine residue in place of valine (**Figure S1**). Non-functional mutations of ClosTE (ClosTE-H171A) and GeoTE (GeoTE-H173A) were generated in order to provide negative controls that account for protein production effects. ClosTE, GeoTE, ClosTE-H171A, and GeoTE-H173A were cloned into pBAD18 and tested in strain RL08ara. Both non-functional bacterial thioesterases produced fatty acid profiles similar to BTE-H204A-expressing cells (**Figure S4**). ClosTE expressing cells did not increase C_8_–C_14_ fatty acid titers significantly (36±1 mg/L from 12±0 mg/L) and was therefore not employed in subsequent studies.

Expression of GeoTE produced highly elevated levels of 8∶0, 10∶1, 10∶0, 12∶1, 12∶0, 14∶1, and 14∶0 fatty acids and detectable levels of odd-chain 9∶0, 11∶0, and 13∶0 fatty acids (**Figure S4**). β-hydroxylated octanoic, decanoic, dodecanoic, tetradecanoic, and hexadecanoic species were also detected according to their elution time patterns, molecular ions, and primary *m/z* ion of 103 (data not shown). Smaller tridecanone and pentadecanone peaks were also identified from their mass spectra (data not shown). These compounds were likely generated by spontaneous decarboxylation following hydrolysis of 3-oxotetradecanoyl-ACP and 3-oxohexadecanoyl-ACP, similar to observations by Goh and coworkers [8] by overexpression of *E. coli* FadM. Elevated levels of 16∶1 but decreased levels of 16∶0, 18∶1, and 18∶0 were observed in cells expressing GeoTE compared with cells expressing GeoTE-H173A. Titers of C_8_–C_14_ fatty acids were 248±55 mg/L compared with 12±9 mg/L in GeoTE-H173A-expressing cultures, and the proportion of C_8_–C_14_ species that were unsaturated was 55 percent, compared with a typical value in BTE-expressing cultures of 23 percent. As a result of this high level of medium-chain length fatty acid production with a higher percentage of unsaturated species than BTE, GeoTE was selected for further characterization of its impact on membrane fatty acid content.

### Unsaturated long-chain fatty acid biosynthesis in BTE and GeoTE expressing cultures

**Figure 3:**
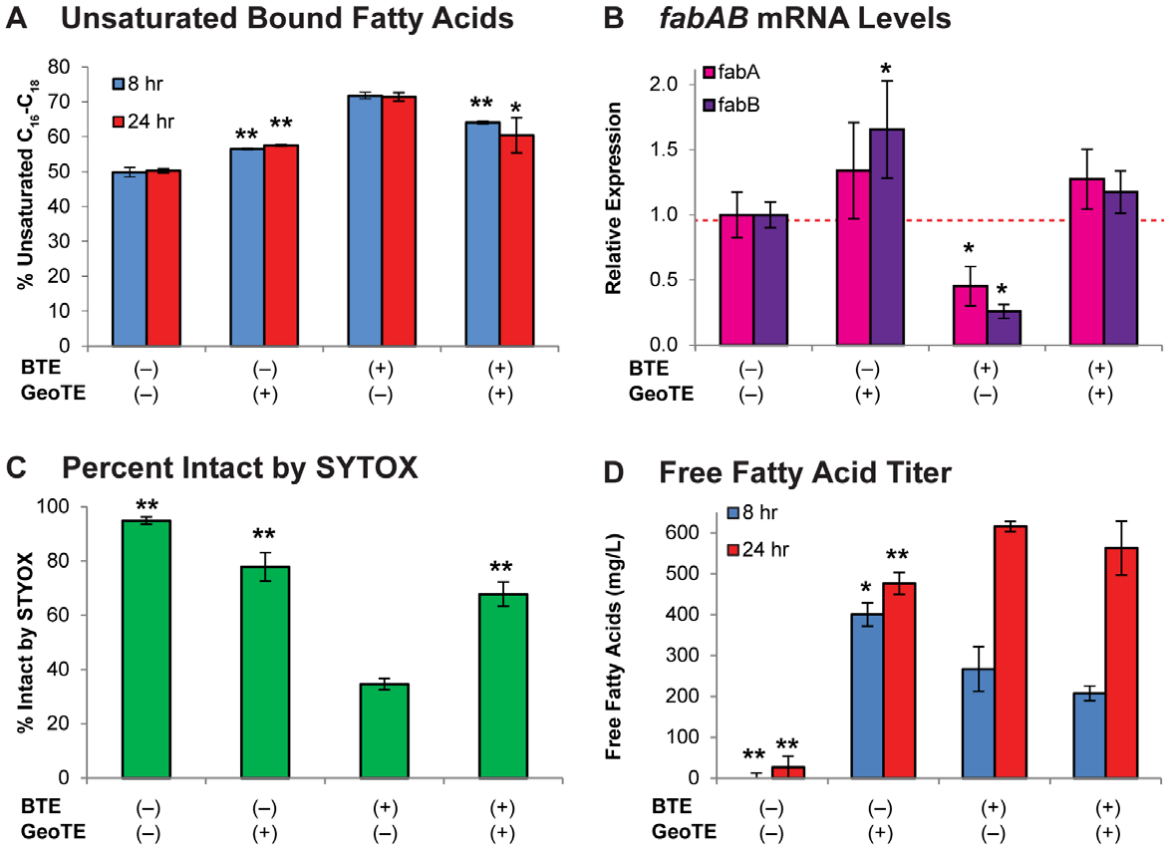
Modulating *E. coli* membrane content via co-expression of saturated and unsaturated acyl-ACP targeting thioesterases. a.) The percentage of unsaturated C_16_–C_18_ and cyclopropane (C_17Δ_) fatty acids were calculated from FAMEs made by base-catalyzed methylation of fatty acids extracted from cultures expressing combinations of BTE and GeoTE at 8 h and 24 h post-inoculation. GeoTE-expressing cells (GeoTE+ BTE−) and GeoTE/BTE co-expressing cells (GeoTE+ BTE+) have a reduced unsaturated content relative to BTE-expressing cells (BTE+ GeoTE−). Error bars represent standard deviations about the mean of biological triplicate samples. * = P-value<0.05, ** = P-value<0.01 compared to BTE^+^ GeoTE^−^ cultures at the same sampling time. b.) Transcript levels of *fabA* and *fabB* determined by qPCR on samples harvested 1 hour hour post-induction were normalized to BTE− GeoTE− (RL08ara pBAD33-BTE-H204A pBAD18-GeoTE-H173A) samples. Levels of *fabA* and *fabB* were decreased in cells expressing only BTE. Conversely, levels of *fabA* or *fabB* were statistically the same or higher in cells expressing GeoTE or GeoTE and BTE. Error bars represent propagated standard errors about the mean of biological triplicate samples. * = P-value<0.05 for C_q_ values compared against *fabA* or *fabB* in cultures expressing only non-functional thioesterases (BTE^−^ GeoTE^−^). c.) The percentage of intact cells were calculated from histograms of cells stained with SYTOX Green 8 h post-inoculation. Cultures expressing only BTE were the least intact. Cultures expressing only GeoTE, and both GeoTE and BTE were over 50% intact. Error bars represent standard errors about the mean of biological triplicate samples. ** = P-value<0.01 compared to BTE^+^ GeoTE^−^. d.) FFA titers from strains expressing combinations of BTE and GeoTE. FFA titers were determined at 8 and 24 h post-inoculation. Cultures expressing only GeoTE exhibited the highest titer at 8 h, with a minor increase observed after 24 h. Nearly equivalent titers were reached after 24 h in cultures expressing only BTE, or co-expressing BTE and GeoTE. Error bars represent standard deviations about the mean of biological triplicate samples. * = P-value<0.05, ** = P-value<0.01 compared to BTE^+^ GeoTE^−^ cultures at same sampling time.

The effect of GeoTE expression and GeoTE/BTE co-expression on membrane lipid composition was compared to that of BTE expression. Analysis of bound fatty acids by base-catalyzed methylation revealed that cultures co-expressing GeoTE and BTE-H204A, as well as cultures co-expressing GeoTE and BTE, maintained a lower percentage of C_16_–C_18_ unsaturated fatty acids than cultures co-expressing BTE and GeoTE-H173A (with P<0.05 for all values) and a similar percent unsaturation to negative control cultures co-expressing BTE-H204A and GeoTE-H173A (**Figure 3a**). These trends were consistent with the anticipated highest depletion of saturated acyl-ACPs by BTE, followed by BTE and GeoTE co-expression, followed by GeoTE alone. Interestingly, GeoTE and BTE co-expressing cultures exhibited the highest percentage of bound fatty acids as cyclic C_17_.

If expression of GeoTE, or co-expression of GeoTE and BTE resulted in a shift toward a larger ratio of saturated to unsaturated acyl-ACPs, it would be expected that *fabA* and *fabB* levels would be restored to higher levels than in cultures expressing only BTE. The levels of *fabA* and *fabB* transcript were quantified by qPCR from biological triplicate cultures of each strain (**Figure 3b**). Indeed, in both the GeoTE/BTE-H204A and GeoTE/BTE co-expressing cultures, levels of *fabA* and *fabB* were restored to levels present in the control culture (co-expressing BTE-H204A and GeoTE-H173A), and were higher than levels present in the BTE/GeoTE-H173A cultures. Expression of *fabB* was higher with statistical significance (P<0.05) in the GeoTE/BTE-H204A expressing cultures than in the GeoTE-H173A/BTE-H204A expressing cultures, indicating that expression of a thioesterase with this degree of unsaturated acyl-ACP substrate specificity resulted in a lower amount of FabR repression than is present in wild-type cells. The fact that expression of *fabA* was not higher with statistical significance was also consistent within this framework, as *fabA* is repressed less strongly, and by analogy also de-repressed less strongly, by FabR than *fabB* due to weaker binding of FabR within the promoter region upstream of *fabA* relative to the promoter region upstream of *fabB* [17].

### Cell growth and viability analysis of GeoTE and BTE expressing cultures

SYTOX Green staining and flow cytometry analysis of cell samples collected 8 h post-inoculation revealed differences in the percentage of intact cells between strains expressing the two thioesterases (**Figure 3c**). BTE-H204A and GeoTE-H173A expressing cells exhibited a typical non-functional thioesterase expressing value of 94.8±1.3 percent intact, while cells expressing only functional BTE were 34.6±2.1 percent intact. Expression of only functional GeoTE, however, resulted in a higher percentage of intact cells (77.8±5.2 percent), while co-expression of functional GeoTE and BTE resulted in 67.7±4.5 percent intact cells. These data were consistent with the intermediate C_16_ to C_18_ unsaturated fatty acid content of the dual thioesterase expressing strain.

A percent viable cells analysis obtained by dividing CFU mL^−1^ by the number of flow cytometry events measured per mL of original cell culture generally followed trends in the SYTOX Green data for percent intact cells (**Table 2**). While non-FFA overproducing BTE-H204A and GeoTE-H173A expressing cells were 90±14 percent viable, BTE and GeoTE-H173A expressing cells were only 5±1 percent viable, and BTE-H204A and GeoTE expressing cells were 45±12 percent viable. In contrast to the SYTOX Green assay, BTE and GeoTE expressing cells were only 3±2 percent viable. As we have observed previously [11], plated cells from BTE-expressing cultures exhibited a wide variety of different colony sizes after one night incubation at 37°C, and one additional night incubation at room temperature was required for all colonies to become visible. Plated cells from GeoTE-expressing cultures exhibited an even larger distribution of colony sizes, with new colonies appearing after 1 night at 37°C and 4 nights at room temperature. BTE plus GeoTE expressing cultures similarly required a total of 5 days before new colonies stopped appearing. Therefore it appeared that large percentages of strains expressing one or both thioesterases are non-lysed but also non-culturable on LB agar.

**Table 2:** Viability analysis of strains expressing combinations of BTE and GeoTE.

BTE	GeoTE	Flow Cytometer (events mL^−1^)	Plate Counts (CFU mL^−1^)	Normalized CFU (CFU event^−1^)
(−)	(−)	(5.75±0.43)×10^9^	(5.2±0.7 )×10^9^	0.90±0.14
(−)	(+)	(4.93±0.15)×10^9^	(2.20±0.60)×10^9^	0.45±0.12
(+)	(−)	(3.61±0.50)×10^9^	(1.9±0.4 )×10^8^	0.05±0.01
(+)	(+)	(1.37±0.76)×10^9^	(4.0±1.6 )×10^7^	0.03±0.02

Cultures of RL08ara harboring combinations of pBAD33-BTE-H204A (BTE^−^) or pBAD33-BTE (BTE^+^), and pBAD18-GeoTE-H173A (GeoTE^−^) or pBAD18-GeoTE (GeoTE^+^) grown in LB+0.4% glycerol and antibiotics at 37°C for 8 hours. Reported values are forward scatter triggered flow cytometry events per mL original culture volume of SYTOX Green stained cells, plate counts (CFU mL^−1^) after 5 days incubation, and plate counts normalized to flow cytometry events (CFU event^−1^), as another estimate of percentage live cells.

### Fatty acid production of GeoTE and BTE expressing cultures

After 8 h growth, cultures expressing only functional GeoTE exhibited a higher total fatty acid titer (513±12 mg L^−1^) than cultures expressing only functional BTE (364±42 mg L^−1^) (**Figure 3d**). This higher productivity could be due to a larger percentage of viable, intact cells in these cultures (**Table 2**). Cultures expressing both functional thioesterases produced 255±117 mg L^−1^ after 8 hours, reflecting the lower cell counts and reduced growth in these cultures. After 24 hours, titers for GeoTE, BTE, and both GeoTE and BTE expressing cultures reached 569±8, 651±57, and 653±60 mg L^−1^ respectively (**Figure 3d**). The fatty acid composition of cultures expressing both thioesterases was intermediate between cultures expressing only functional GeoTE or BTE.

## Discussion

In prior work, we observed cell lysis, decreased viability, increased membrane unsaturated fatty acid content, and transcriptional evidence of membrane stresses, cell depolarization, and impaired aerobic respiration when BTE was expressed in *E. coli* [11]. The present study correlated cell lysis and decreased cell viability with elevated unsaturated fatty acid content in the membrane, suggesting a causative relationship in strains producing FFA. Achieving the maximum theoretical yield of FFAs (as determined by constraint-based modeling) requires aerobic respiration and the use of the membrane-bound transhydrogenase PntAB, and therefore maintenance of inner cell membrane integrity and properties. To develop commercial strains for advanced biofuel applications, it is critical to maintain necessary active metabolic pathways throughout the course of production and to understand the source of disruptions to these pathways. Furthermore, it is important to not induce selective pressures that favor loss of endogenous FFA production. In the last five years, many groups have implemented strategies for producing FFA that are based on expression of acyl-ACP thioesterases in *E. coli*. In these studies, a wide range of titers (and correspondingly, yields in batch cultures) have been reported when different thioesterases were expressed [3]. In most cases there is no clear rationale to explain the differences in observed titers. We postulate that the degree to which a thioesterase alters the long-chain acyl-ACP pool destined for incorporation in membrane phospholipids is one source of varying titers reported in different engineered FFA-overproducing strains. For example, this hypothesis may explain the results of Lu et al. [29], wherein co-expression of a cytosolic form of *E. coli* TesA (thioesterase I) and the plant FatB-type thioesterase from *Cinnamomum camphorum* (CcTE) produced higher titers than expression of CcTE alone. The substrate specificity for CcTE is most enriched in saturated C_14_ [30], whereas TesA has a broad C_12_–C_18_ specificity that is highest toward unsaturated C_16_ and C_18_ fatty acids [1]. High titers were also reported from a strain designed to undergo reversal of β-oxidation and overexpressing an acyl-CoA thioesterase (*E. coli* FadM) [31], may be due to this pathway being independent from fatty acid biosynthesis and therefore lacking impacts to membrane-bound fatty acids such as those imposed by acyl-ACP thioesterase expression. If true, this hypothesis motivates further work to control membrane content independent of thioesterase specificity.

While alteration of thioesterase substrate specificity by expression of GeoTE succeeded in restoring membrane unsaturated fatty acid content and reducing cell lysis, it resulted in a more diverse product profile. This outcome may be acceptable when fuels or other chemical mixtures are targeted, but not when specific oleochemicals are targeted. This study further underscored the criticality of FabR in maintaining membrane integrity in thioesterase-expressing cells, and strongly suggests that the level of FabR repression is insufficient in *fabR* ^+^ BTE-expressing cultures, possibly due to cells being unable to down-regulate *fabA* and *fabB* to a sufficient degree to counter the effects on the acyl-ACP pool resulting from BTE expression. While overexpression of FabR resulted in severe impacts toward growth, a more promising strategy to pursue in the future entails promoter engineering of P*_fabA_* and P*_fabB_* to achieve increased repression by FabR.

## Conclusion

The unsaturated fatty acid content of the membrane is a key parameter that influences cell lysis under conditions of endogenous FFA production. While FabR is a major controller, its level of repression appears to be insufficient to prevent elevated unsaturated acyl-ACP pools as a result of expression of BTE, which hydrolyzes primarily saturated acyl-ACPs. The current data cements the relationship between acyl-ACP thioesterase expression and membrane unsaturated fatty acid content. It was shown that acyl-ACP pools can be modulated by thioesterase selection. Expression of a thioesterase with primarily unsaturated acyl-ACP substrate specificity (GeoTE) reduced membrane unsaturated fatty acids, increased cell viability, reduced lysis, and increased FFA productivity during the first 8 hours of growth. Further strategies geared toward modulation of membrane lipid content for improved cellular fitness are being pursued that do not result in alteration of the FFA product profile, such as promoter engineering of FabR binding sites upstream of *fabA* and *fabB*.

## Supporting Information

Figure S1 **Synthetic codon-optimized gene sequences for the acyl-ACP thioesterases from** ***Geobacillus*** **Y412MC10 (GeoTE) and** ***Clostridium thermocellum*** **(ClosTE).** The location of the H173A mutation in GeoTE is shown in underlined italics and involved substitution of CAT with GCT. The location of the H171A mutation in ClosTE is shown in underlined italics and involved substitution of CAC with GCC. Restriction sites (for XmaI and HindIII) used for cloning are underlined. The ribosome binding site is shown in blue, and the spacer sequence is shown in red. Start and stop codons are bolded.
(DOC)

Figure S2 **Alignment of amino acid sequence of BTE with bacterial acyl-ACP thioesterases from** ***Geobacillus*** **Y412MC10 (GeoTE) and** ***Clostridium thermocellum*** **(ClosTE).** The catalytic histidine at position 204 in BTE and HVNN motif aligns with histidine-173 in GeoTE and histidine-171 in ClosTE. Valine-205 in BTE aligns with a similar leucine residue in GeoTE.
(TIF)

Figure S3 **Comparative analysis of acid and base catalyzed FAME preparations from cultures expressing BTE and GeoTE-H173A.** Cultures were sampled 8 h and 24 h post-inoculation. Free fatty acids can be calculated by subtracting the base titers from acid titers. BTE hydrolyzes predominantly 12∶1, 12∶0, 14∶1, and 14∶0 fatty acids, and does not appreciably hydrolyze C_16_–C_18_ fatty acids. GeoTE hydrolyzes a wide range of C_8_ to C_16_ fatty acids, with the highest activity towards 12∶1, 12∶0, 14∶1, 14∶0, 16∶1, and 16∶0 species.
(TIF)

Figure S4 **Total fatty acid analysis of functional and mutagenized GeoTE and ClosTE.** Cultures were grown for 24 hours in LB plus 0.4% glycerol at 37°C. Expression of GeoTE resulted in high titers of overexpressed FFAs with a diverse profile and 12∶1 as the highest concentration product. Expression of ClosTE produced low FFA titers.
(TIF)

Table S1 **Oligonucleotide primers used in this study.**
(DOC)
