# Supplementary material for: Modulating Membrane Composition Alters Free Fatty Acid Tolerance in Escherichia coli
Source: PLoS One. 2013 Jan 21;8(1):e54031. doi: 10.1371/journal.pone.0054031 (PMC3549993; doi:10.1371/journal.pone.0054031)
Supplement: Figure S1 — Synthetic codon-optimized gene sequences for the acyl-ACP thioesterases from Geobacillus Y412MC10 (GeoTE) and Clostridium thermocellum (ClosTE). The location of the H173A mutation in GeoTE is shown in underlined italics and involved substitution of CAT with GCT. The location of the H171A mutation in ClosTE is shown in underlined italics and involved substitution of CAC with GCC. Restriction sites (for XmaI and HindIII) used for cloning are underlined. The ribosome binding site is shown in blue, and the spacer sequence is shown in red. Start and stop codons are bolded. (DOC) [file pone.0054031.s001.doc]

**GeoTE synthesized gene sequence**

1 CCCGGGAAGG AGGTATATAA A**ATG**GAGCTG ATGATCGATA AGTGGACCGA AGAATATACC

61 ATTCAGAGCG TTGATGCCGA TTTTAAAGGT GATTGTCGTT GGAGCAGCCT GCTGAGCATT

121 CTGCAACGTG CAGCAGATCG TCATATTGAA GCACTGGGTA TTAGCCGTGA AGAAATGATT

181 GAACGTGGTA TGGGTTGGAT GCTGATTACC CTGGAACTGG AAATGCGTCG TATGCCTCGT

241 GATATGGAAA ATGTTTATGT TGATACCTGG TCACGTGGTA GCAAAGGTGC ACTGTGGCAT

301 CGTGATTATC GTATCAAAAA TGGTGATGGT GAACTGCTGG GTGAAGGTCG TAGCGTTTGG

361 GCACTGGTTG ATATTCATAA ACGTAAAATT CTGCGTCCGA GCATGTTTCC GTATGAAGTT

421 CCGATTGGTC AAGAAACCGT TGGCGAACTG CCGAGCAAAG CAGTTCTGCC GGAAGGTGTT

481 CAACTGGATG ATGCATATAC CTATAGCGTT CGTTATAGCG GCATTGATAC CAATGGT*CAT*

541 CTGAATAATG CACGTTATGC CGATCTGTGT TTTGATGTTC TGGATGAACA AGAACTGCGT

601 GAAGGTCTGG TTACCGGTTT CAAAATTACC TATCTGAATG AAGCCCGTCT GAAAGATACC

661 ATGCTGATCA AACGTAGTGC CGAAGAAAAT AATCGTGTTT ATGTGCAGGG CACCAGTCCG

721 GATGGCACCA ACTTTTTTGA AGCAGCAATT GTTCGTGAAA GCCATCATCA CCATCACCAC

781 **TAA**GCTT

**ClosTE synthesized gene sequence**

1 CCCGGGAGGA GGTAAATTAA **ATG**CAGAAAA AACGCTTCAG CAAAAAATAC GAGGTGCACT

61 ATTATGAGAT CAACAGTATG CAAGAAGCAA CCCTGCTGAG CCTGCTGAAT TATATGGAAG

121 ATTGTGCAAT TAGCCATAGC ACCAGCGCAG GTTATGGTGT TAATGAACTG CTGGCAGCAG

181 ATGCAGGTTG GGTTCTGTAT CGTTGGCTGA TTAAAATCGA TCGTCTGCCG AAACTGGGTG

241 AAACCATTAC CGTTCAGACC TGGGCAAGCA GCTTTGAACG TTTTTATGGT AACCGCGAGT

301 TTATTGTTCT GGATGGTCGT GATAATCCGA TTGTTAAAGC CAGCAGCGTG TGGATCTATT

361 TTAACATCAA AAAACGTAAA CCGATGCGCA TTCCGCTGGA AATGGGTGAT GCCTATGGTA

421 TTGATGAAAC CCGTGCACTG GAAGAACCGT TTACCGATTT TGATTTCGAT TTTGAGCCGA

481 AAGTGATCGA AGAGTTTACC GTTAAACGTA GCGATATTGA TACCAACAGC *CAC*GTGAACA

541 ACAAAAAATA CGTGGATTGG ATTATGGAAA CCGTTCCGCA GCAGATCTAT GATAACTATA

601 AAGTTACCAG CCTGCAGATC ATCTACAAAA AAGAAAGCAG CCTGGGTAGC GGTATTAAAG

661 CAGGTTGTGT GATTGATGAA CAGAACACCG ATAATCCGCG TCTGCTGCAT AAAATCTGGG

721 ATAAAAACAC CGGTCTGGAA CTGGTTAGCG CAGAAACCAT TTGGCAGAAA ATTCAGAGCC

781 ATCATCACCA TCACCAC**TAA** GCTT

**Figure S1.** Synthetic codon-optimized gene sequences for the acyl-ACP thioesterases from *Geobacillus* Y412MC10 (GeoTE) and *Clostridium thermocellum* (ClosTE). The location of the H173A mutation in GeoTE is shown in underlined italics and involved substitution of CAT with GCT. The location of the H171A mutation in ClosTE is shown in underlined italics and involved substitution of CAC with GCC. Restriction sites (for XmaI and HindIII) used for cloning are underlined. The ribosome binding site is shown in blue, and the spacer sequence is shown in red. Start and stop codons are bolded.
