# Supplementary figures and images for: Modulating Membrane Composition Alters Free Fatty Acid Tolerance in Escherichia coli
Source: PLoS One. 2013 Jan 21;8(1):e54031. doi: 10.1371/journal.pone.0054031 (PMC3549993; doi:10.1371/journal.pone.0054031)

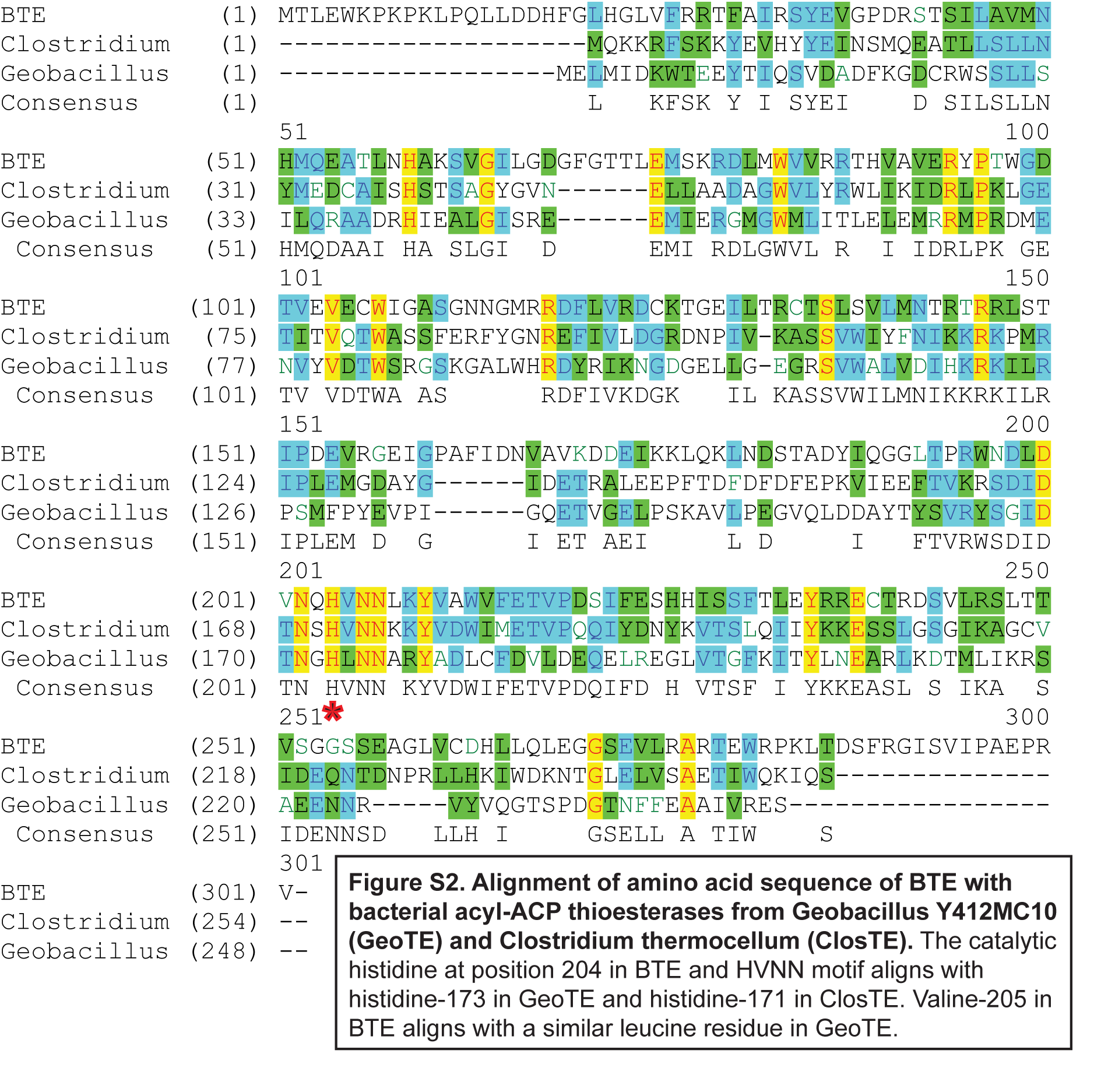

Supplement: Figure S2 — Alignment of amino acid sequence of BTE with bacterial acyl-ACP thioesterases from Geobacillus Y412MC10 (GeoTE) and Clostridium thermocellum (ClosTE). The catalytic histidine at position 204 in BTE and HVNN motif aligns with histidine-173 in GeoTE and histidine-171 in ClosTE. Valine-205 in BTE aligns with a similar leucine residue in GeoTE. (TIF) [file pone.0054031.s002.tif]

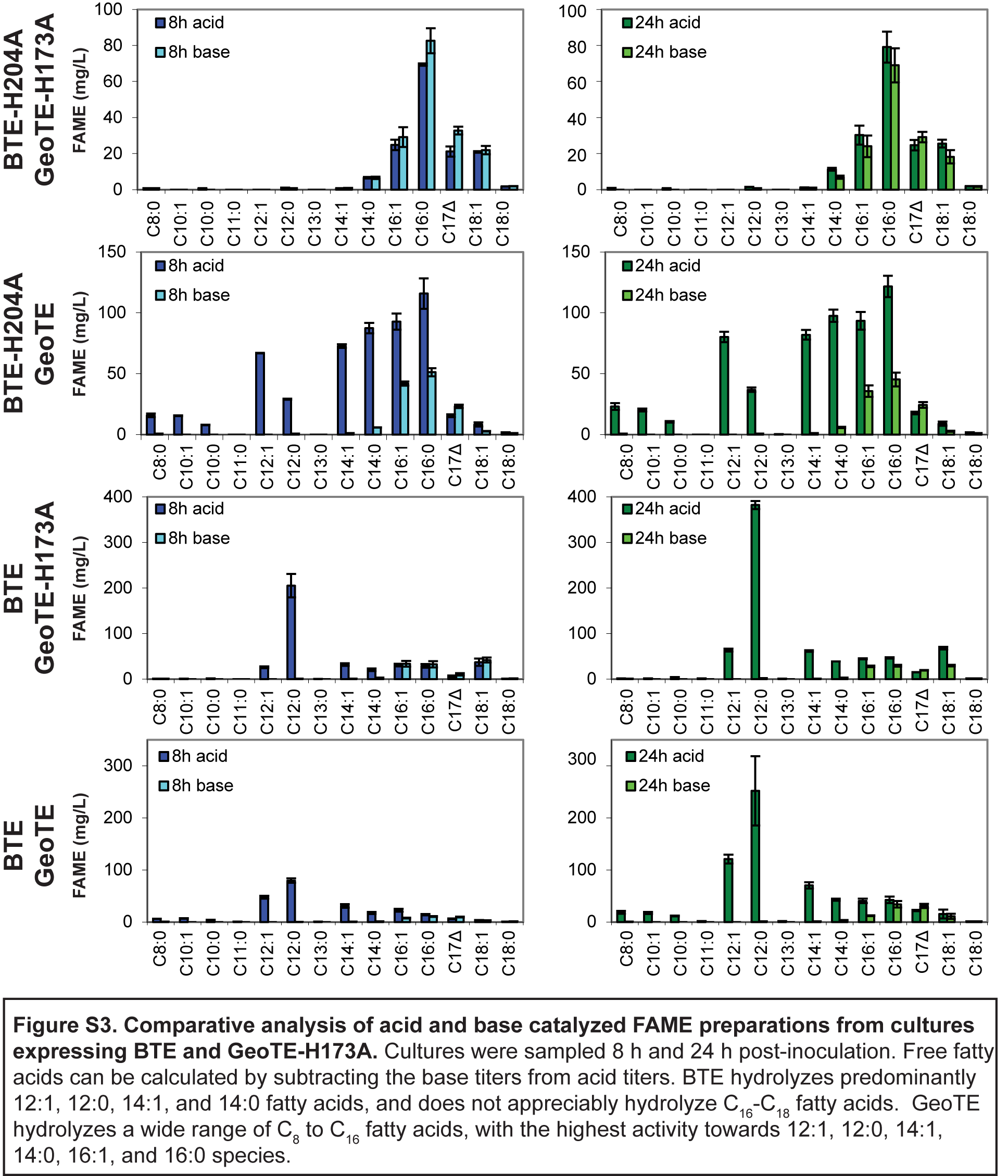

Supplement: Figure S3 — Comparative analysis of acid and base catalyzed FAME preparations from cultures expressing BTE and GeoTE-H173A. Cultures were sampled 8 h and 24 h post-inoculation. Free fatty acids can be calculated by subtracting the base titers from acid titers. BTE hydrolyzes predominantly 12∶1, 12∶0, 14∶1, and 14∶0 fatty acids, and does not appreciably hydrolyze C16–C18 fatty acids. GeoTE hydrolyzes a wide range of C8 to C16 fatty acids, with the highest activity towards 12∶1, 12∶0, 14∶1, 14∶0, 16∶1, and 16∶0 species. (TIF) [file pone.0054031.s003.tif]

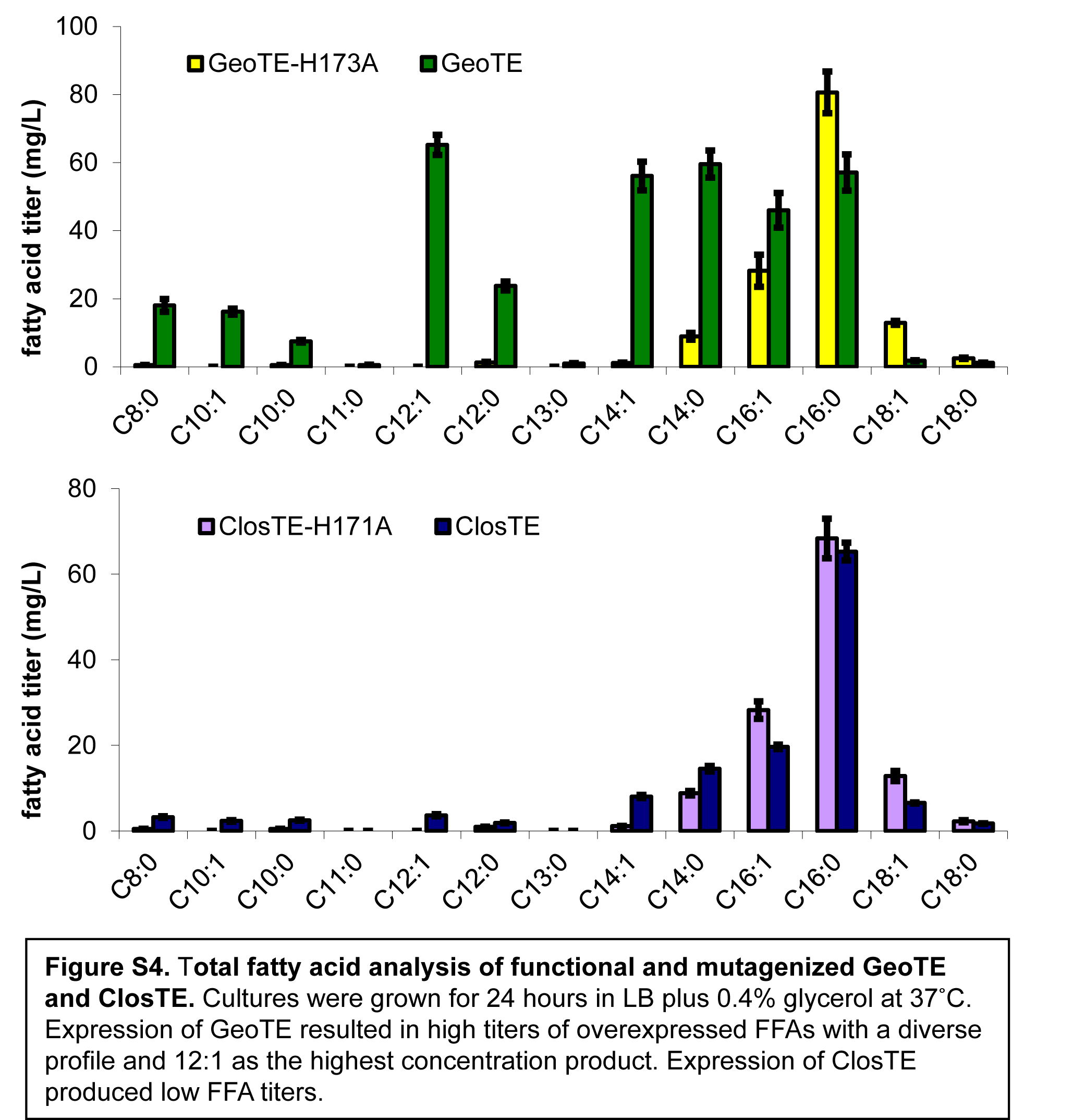

Supplement: Figure S4 — Total fatty acid analysis of functional and mutagenized GeoTE and ClosTE. Cultures were grown for 24 hours in LB plus 0.4% glycerol at 37°C. Expression of GeoTE resulted in high titers of overexpressed FFAs with a diverse profile and 12∶1 as the highest concentration product. Expression of ClosTE produced low FFA titers. (TIF) [file pone.0054031.s004.tif]
