# Supplementary material for: Modulating Membrane Composition Alters Free Fatty Acid Tolerance in Escherichia coli
Source: PLoS One. 2013 Jan 21;8(1):e54031. doi: 10.1371/journal.pone.0054031 (PMC3549993; doi:10.1371/journal.pone.0054031)
Supplement: Table S1 — Oligonucleotide primers used in this study. (DOC) [file pone.0054031.s005.doc]

**Table S1.** Oligonucleotide primers used in this study.

|  | **Primer name** | **Sequence (5' to 3')**^a,b^ |
| --- | --- | --- |
| 1 | fadD_colPCR_fwd | ACGGCATGTATATCATTTGGG |
| 2 | fadD_colPCR_rev | CTTTAGTGGGCGTCAAAAAAAAC |
| 3 | araBAD_colPCR_fwd | AAGCGGGACCAAAGCCATGAC |
| 4 | araBAD_colPCR_rev | AGGAGACTTCTGTCCCTTGCG |
| 5 | araFGH_colPCR_fwd | GGTACCAAAGACAACAAGGATTTCC |
| 6 | araFGH_colPCR_rev | CTATACTTACATGTCTGTAAAGCGCG |
| 7 | ParaE_colPCR_fwd | CATGGCGACCAACAATACTC |
| 8 | ParaE_colPCR_rev | TTCCGCCTCAATATGACG |
| 9 | fabR_colPCR_fwd | GTACGTAAAAGAACCGGCCAAAG |
| 10 | fabR_colPCR_rev | GCTGCTGCTGGCGTTAGTTG |
| 11 | pBAD33-C280*_fwd | GGGCTCGAGTTAACCGGCACGGAACTCGCTCG |
| 12 | pBAD33-C280*_rev | GGGCTCGAGTTGGTAACGAATCAGACAATTGACGGC |
| 13 | GeoTE_fwd | GCGCCCGGGAAGGAGGTATATAAAATGG |
| 14 | GeoTE-His_rev | GGGAAGCTTAGTGGTGATGGTGATGATGGCTTTCACGAACAATTGCTGCTTCAAA |
| 15 | ClosTE_fwd | GCGCCCGGGAGGAGGTAAATTAAATGC |
| 16 | ClosTE-His_rev | GGGAAGCTTAGTGGTGATGGTGATGATGGCTCTGAATTTTCTGCCAAATGGTTTC |
| 17 | fabR_RBSeng_fwd | GCGCCCGGGAATCATACCCCATCGGTACCCATAGGTAGGTTCACATGTTCATTCTCTGGTATAGTGCC |
| 18 | fabR_rev | GGGTCTAGATTACTCGTCCTTCACATTTCCCG |
| 19 | fabA_qPCR_fwd | CACTTTATTGGCGATCCGGTTATG |
| 20 | fabA_qPCR_rev | CTTTACCTTCGCCGCCCAGC |
| 21 | fabB_qPCR_fwd | ATGCATGGCGTTGATACCCCAATC |
| 22 | fabB_qPCR_rev | AACACTTCACGGATAGCTGCCAG |
| 23 | fabR_qPCR_fwd | ATTGCGGAACTTGCGGACTATCTG |
| 24 | fabR_qPCR_rev | TGCCGACGTTGTTCGACGCC |
| 25 | acpP_qPCR_fwd | GCGGATTCTCTTGACACCGTTG |
| 26 | acpP_qPCR_rev | TGATGTAATCAATGGCAGCCTGAAC |
| ^a^ Primers containing 'qPCR' in the name were used for amplification of cDNA in quantitative PCR reactions.  Primers containing 'colPCR' were used colony PCR verification of chromosomal gene insertions and deletions.  Primers containing restriction sites were used for amplification of insertions for cloning. | | |
| ^b^ Restriction sites are underlined | | |
